# Surgical Treatment of Metastatic Bone Disease—When Decisions at End-of-Life Really Makes the Difference

**DOI:** 10.3390/cancers13112581

**Published:** 2021-05-25

**Authors:** Michala Skovlund Sørensen, Michael Mørk Petersen

**Affiliations:** 1 Musculoskeletal Tumor Section, Department of Orthopedics, Rigshospitalet, University Hospital of Copenhagen, Inge Lehmanns Vej 6, DK-2100 Copenhagen Ø, Denmark; 2 Department of Clinical Medicine, Faculty of Health and Medical Sciences, University of Copenhagen, DK-2200 Copenhagen N, Denmark

The current era within the field of surgical treatment of metastatic bone disease (MBD) is best described as the beginning of a paradigm shift. We are moving away from the thought of that all the patients are at their very terminal stage when they present with a bony lesion in need of surgical intervention, and thus treating them with a surgical solution inducing a minimal surgical trauma, to treatment modalities aiming to preserve or improve the quality of life, or even for some selected cases, hoping to prolong survival by performing metastasectomy with a tumor-free surgical margin [1,2].

The surgical treatment of MBD is a challenging field as it is does not only demand a multidisciplinary approach, often including oncology, radiology, pathology, and orthopedic surgeons but often requires a specialized spine surgeon or an orthopedic oncologist, in order to address the patient and their disease before selecting the optimal surgical treatment modality.

As such, the orthopedic surgeon, subspecialized into surgical oncology or not, must move away from the thought of MBD being something one needs to fix quickly before the patient succumbs to the disease, to a condition that might require a multidisciplinary approach in order to address: (1) if the treatment strategy should aim for local control or systemic control (2) what implant is most likely to outlive the patient (minimizing the risk of implant failure) (3) if the patient is fit for the surgical choice (hence does the surgery pose a risk for survival of the most fragile patients). These questions must be asked in the clinic prior to choosing the treatment (surgical or not), but are also most certainly important research questions that need to be addressed in the scientific literature.

In order to standardize the prediction of survival, several researchers have attempted to develop prediction models for the survival of MBD patients [3,4,5,6,7]. Validations of these models are important, to assure the models will also work for patients treated in other facilities outside the original study population, but also in the new era where new treatment modalities might increase the expected survival for the different primary cancers. The models consist of variables where the majority is dependent upon the attending physician, thus making them woundable to bias. Additionally, due to continuous improvement in oncological treatment, MBD patients are expected to live with a disseminated disease for a longer time. This, and changes over time, demand that these models are continuously refitted and validated to be useful in the daily clinical work.

Secondly, there is a need for improved evidence for implant survival stratified for anatomical location, primary tumor, adjuvant therapy and improved statistical analysis, taking death into account as a competing risk for implant failure. Historically, Kaplan–Meier analysis has been used to address the time-dependent risk of implant failure, and as it has been expressed as a concern in studies of orthopedic implants in general; the risk of over-estimating the revision risk is increased when the competing risk of event is an issue [8]. We know from cross-sectional studies of a population that the patients with the poorest survival probabilities are selected to undergo treatment with osteosynthesis and patients with long-term survival expectations to joint replacement surgery [9]. Blindly comparing the implant revision risk between the implants, not taking death as a competing factor into consideration, will inherently result in the overestimation of risk for the osteosynthesis group. However, maybe it is more in the patient’s interest to obtain the expected performance of the implant presented rather than implant failure?

This leads us to our final plea for future studies within the research fields of surgical treatment of MBD. We need to address and acknowledge the inherent bias most studies will have, as randomized controlled trials are difficult if not impossible to conduct due to small incidence, difference in tumor histology and structure, different and often short patient survival, and substantial comorbidity, etc. Additionally, we need to underline the limitations the study design draws to the conclusion and admit that sometimes, good clinical practice is the highest reachable level of evidence, and act accordingly hereto.

In spite of these potential study limitations, we need to overcome these uncertainties and create new and more precise, unbiased prediction models (objective prognostic markers), the validation of prediction models, the investigation of quality of life using patient reported outcome measures and to evaluate the performance of implants in a prospective setting, and last but not least—to try to answer the question—when (if ever) is metastasectomy with a tumor-free surgical margin indicated in patients suffering from MBD? In our experience, one should always consider metastasectomy with a tumor-free surgical margin in patients with solitary metastatic lesions of the extremities, even though the reconstruction will then require a tumor prosthesis, and for pelvis tumors, sometimes even a custom-made pelvic implant, because this surgical approach has been shown to increase the probability of survival by two to three times in kidney cancer patients with a solitary metastatic lesion of the extremities [1,2].
